# Diffusion-controlled crack propagation in alkali feldspar

**DOI:** 10.1007/s00269-018-0983-9

**Published:** 2018-07-07

**Authors:** E. Petrishcheva, M. Rieder, J. Predan, F. D. Fischer, G. Giester, R. Abart

**Affiliations:** 1 Department of Lithospheric Research, University of Vienna, 1090 Vienna, Austria; 2 Faculty of Mechanical Engineering, University of Maribor, Maribor, Slovenia; 3 Institute of Mechanics, Montanuniversität Leoben, 8700 Leoben, Austria; 4 Institute of Mineralogy and Crystallography, University of Vienna, 1090 Vienna, Austria

## Abstract

The chemically driven propagation of interacting parallel cracks in monoclinic alkali feldspar was studied experimentally. Single crystals of potassium-rich gem-quality sanidine were shifted towards more sodium-rich compositions by cation exchange with a NaCl–KCl salt melt at a temperature of $850\,^{\circ }\hbox {C}$ and close to ambient pressure. Initially, a zone with elevated sodium content formed at the crystal surfaces due to the simultaneous in-diffusion of sodium and out-diffusion of potassium, where the rate of cation exchange was controlled by sodium–potassium interdiffusion within the feldspar. A chemical shift of potassium-rich alkali feldspar towards more sodium-rich compositions produces highly anisotropic contraction of the crystal lattice. This induced a tensile stress state in the sodium-rich surface layer of the crystals, which triggered the formation of a system of nearly equi-spaced parallel cracks oriented approximately perpendicular to the direction of maximum shortening. Crack propagation following their nucleation was driven by cation exchange occurring along the crack flanks and was controlled by the intimate coupling of the diffusion-mediated build-up of a tensile stress state around the crack tips and stress release by successive crack propagation. The critical energy release rate of fracturing was determined as 1.8–2.2 $~ \hbox {J}\,\hbox {m}^{-2}$ from evaluation of the near-tip J-integral. The mechanism of diffusion-controlled crack propagation is discussed in the context of high-temperature feldspar alteration.

## Introduction

Feldspar is the most abundant mineral in the Earth’s crust. Rock-forming feldspar pertains to the ternary solid solution among the phase components albite ($\mathrm{NaAlSi}_{3}\mathrm{O}_8$), K-feldspar ($\mathrm{KAlSi}_3\mathrm{O}_8$), and anorthite ($\mathrm{CaAl}_2\mathrm{Si}_2\mathrm{O}_8$). The $\mathrm{Si}^{4+}$ and $\mathrm{Al}^{3+}$ cations are tetrahedrally coordinated by oxygen, and the corner-sharing $\mathrm{SiO}_4$ and $\mathrm{AlO}_4$ tetrahedral form a three-dimensional network (Ribbe 1983). The alkali and alkaline earth cations are located in large cavities within the tetrahedral framework and are coordinated by six ($\hbox {Na}^+$) to nine ($\hbox {K}^+$) oxygen anions. The Si–O and Al–O bonds in the tetrahedral framework are strong, and intracrystalline diffusion of Si and Al is extremely sluggish (Cherniak 2010). In contrast, Na and K are relatively mobile, and intracrystalline diffusion of Na and K as well as Na–K interdiffusion are comparatively fast (Cherniak 2010; Petrishcheva et al. 2014; Schäffer et al. 2014). The high mobility of the alkali cations makes the alkali feldspar prone to chemical re-equilibration. For example, alkali feldspar of intermediate composition tends to exsolve during cooling, which leads to the formation of perthites in slowly cooled magmatic and metamorphic rocks (Yund 1984; Abart et al. 2009; Petrishcheva and Abart 2009, 2012). The lattice parameters of alkali feldspar show considerable compositional dependence. All lattice constants decrease with decreasing potassium mole fraction, $c = \mathrm{K/(K + Na)}$ (molar units), where the effect is most pronounced for the *a*-parameter and comparatively small for the *b*- and *c*-parameters (Kroll and Ribbe 1983; Kroll et al. 1986; Angel et al. 2012). As a consequence, spatial variations of the potassium mole fraction within a single crystal of alkali feldspar produce a state of heterogeneously distributed, non-isostatic stress. When the composition of an alkali feldspar is changed due to interaction with a fluid or melt, the associated stress may exceed the mechanical strength of the feldspar, and fracturing may occur. The newly formed cracks provide pathways for the fluid or melt that brought about the composition change leading to a positive feedback between composition change and fracturing. Replacement of orthoclase (or sanidine) by Na-rich plagioclase is a typical example of high-temperature feldspar alteration accompanying felsic–mafic melt interaction in plutonic and volcanic environments. Chemically induced crack propagation is a viable mechanism for mediating such mineral replacement (Stimac and Wark 1992; Wark and Stimac 1992; Ginibre et al. 2007).

The formation of cracks due to an induced eigenstrain is of pivotal importance in a number of technical applications. Typical situations include eigenstrain caused by temperature- or composition gradients or by phase transformations (Parker 1999). If the associated stresses exceed the material mechanical strength, fracturing occurs, and a wake of fractured material is left behind the propagating thermal, chemical or phase-transformation front. If only one component of the induced stress is tensile, a system of approximately equi-spaced parallel cracks forms, where the characteristic crack spacing depends on the induced stress state (Bazant and Ohtsubo 1977; Bazant et al. 1979; Sumi et al. 1980; Keer et al. 1979).

A similar situation is encountered, when potassium-rich alkali feldspar is shifted towards more sodium-rich compositions by diffusion-mediated cation exchange (Neusser et al. 2012; Scheidl et al. 2013). Neusser et al. (2012) exposed fragments of crushed sanidine and orthoclase crystals to an NaCl–KCl salt melt at $850\,^{\circ }\hbox {C}$ and ambient pressure. Depending on the Na/K ratio of the salt melt, the feldspar crystals were shifted towards more sodium-rich or towards more potassium-rich compositions due to the diffusion-mediated exchange of Na and K between crystal and melt. Irrespective of the direction of the chemical shift, fracturing was observed, whenever the extent of compositional shift exceeded about 15 mole percent. Scheidl et al. (2013) used single crystals of sanidine from the Eifel (Germany) machined to cuboidal plates with either polished (001)- or polished (010)-surfaces. They applied chemical shifts towards more sodium-rich compositions by similar cation-exchange experiments and obtained systems of regularly spaced parallel cracks emanating from the polished (010)- or (001)-surfaces and extending approximately perpendicular to the crystallographic *a*-direction. Based on the notion that the characteristic crack spacing decreases with an increase of the applied compositional shift, the stress intensity factor $K_\mathrm{I}$ for mode I cracks was estimated as $K_\mathrm{Ic} = 2.72\,\mathrm{MPa}\,\mathrm{m}^{1/2}$ and $K_\mathrm{Ic} = 2.30 \,\mathrm{MPa} \,\mathrm{m}^{1/2}$ for cracks emanating from the (001)- and the (010)-surfaces, respectively. Although instructive, the study of Scheidl et al. (2013) is limited due to some simplifying assumptions. It is strictly valid only for isotropic material, and any application to monoclinic alkali feldspar introduces a systematic error. Furthermore, the analysis of Scheidl et al. (2013) is based on the presence of a tensile stress parallel to the free surface. This scenario is representative for crack nucleation, if a uniform chemically altered surface layer exists with its concentration contours parallel to the free surface of the crystal plate. As soon as fracturing occurs and melt penetrates into the interior of the feldspar crystal along the newly formed cracks, the flanks of the cracks serve as new surfaces for cation exchange. Halos of chemically altered feldspar develop along the cracks (Neusser et al. 2012; Scheidl et al. 2013). As a consequence, the eigenstrain and stress fields attain geometries not accounted for in the analysis of Scheidl et al. (2013).

In this study, the simplifying assumptions used by Scheidl et al. (2013) are eliminated. We present the results of time series experiments, where sanidine from Volkesfeld was shifted to more sodium-rich compositions by cation exchange with an NaCl–KCl salt melt at $850\,^{\circ }\hbox {C}$ and ambient pressure. The evolution of the composition and eigenstrain fields around the propagating cracks is explicitly considered in the mechanical analysis. The ABAQUS (https://www.3ds.com/de/produkte-und-services/simulia/produkte/abaqus/) finite element package was used for quantifying the associated stress fields taking full account of the monoclinic symmetry and the elastic- and the diffusion anisotropy of the sanidine. From our analysis, we infer that crack propagation is controlled by a balance between diffusion-mediated Na–K exchange around the crack tip and reduction of the associated stress level by successive crack propagation. The critical energy release rate for crack propagation is quantified by evaluation of the near-tip J-integral.

## Experimental work

### Starting material

**Fig. 1:**
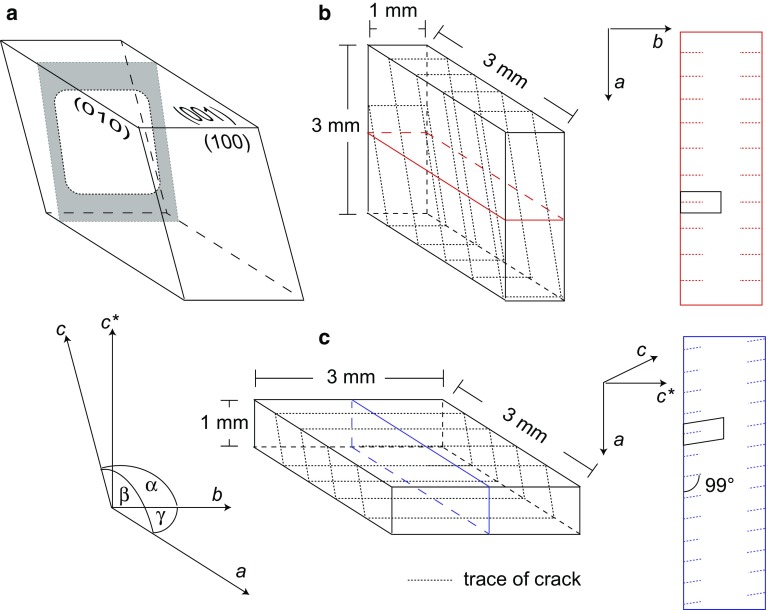
(**a**) Schematic drawing of a parallelepiped consisting of the (100)-, (010)-, (001)-planes of monoclinic feldspar; ${\mathbf {a}}$, ${\mathbf {b}}$ and ${\mathbf {c}}$ are the crystallographic axes, $\alpha = \angle ({\mathbf {b}},{\mathbf {c}}) = 90^\circ$, $\beta = \angle ({\mathbf {a}},{\mathbf {c}}) = 116^\circ$ and $\gamma = \angle ({\mathbf {a}},{\mathbf {b}}) = 90^\circ$; an orthogonal coordinate system *Oxyz* is defined based on ${\mathbf {a}}$, ${\mathbf {b}}$, and ${\mathbf {c}}^\star$, where ${\mathbf {c}}^\star \parallel {\mathbf {a}}\times {\mathbf {b}}$. The grey shaded area indicates the geometry of a crack. The cracks actually extend all around the crystal plate, the segments of the crack emanating from the top and bottom (001)-surface represent the *c*-cracks and the segments of the crack emanating from the left and right (010) surface are the *b*-crack. (**b**) (010)-plate (**c**) (001)-plate; the dashed lines indicate the traces of the cracks on the surfaces of the (001)- and the (010)-plates, the solid blue and red lines show the traces of the cut used for preparing the polished cross-sections for SEM and EPMA analyses; the blue and red rectangles on the far right show the geometry of the cross-sections of a (001)- and a (010)-plate, where the dashed lines indicate the traces of the cracks. The small rectangle and parallelogram indicate the simulation domains used in numerical modelling

Gem-quality alkali feldspar from Volkesfeld (Eifel, Germany) with $\hbox {Or}_{84}\hbox {Ab}_{15}\hbox {Cs}_{01}$ (Demtröder 2011) was used as a starting material. The sanidine is monoclinic with space group C2/m, has a disordered (Al, Si) distribution ($\Sigma \hbox {t}1 = 61$) and is homogenous on the nanometer scale (Neusser et al. 2012). The crystals are optically clear and, apart from the (010)- and (001)-cleavages, they are devoid of cracks or any other flaws. To provide well-defined starting configurations, centimetre-sized transparent sanidine crystals were oriented on a four-circle goniometer and machined to cuboid plates with dimensions 3 $\times$ 3 $\times$ 1 mm. The larger (3 $\times$ 3 mm) surfaces were then polished with diamond paste down to $0.25\,\upmu \hbox {m}$ particle size. Two different specimen orientations with the large (3 $\times$ 3 mm) polished surfaces either parallel to the (001)- or the (010)-lattice planes were produced. Henceforth, the former will be referred to as the (001)-plates and the latter as the (010)-plates. A schematic drawing of the two differently oriented plates and the corresponding tension cracks is shown in Fig. 1. In the following, an orthogonal coordinate system *Oxyz* generated by the vectors ${\mathbf {a}}$, ${\mathbf {b}}$, and ${\mathbf {c}}^\star$, where ${\mathbf {c}}^\star \parallel {\mathbf {a}}\times {\mathbf {b}}$ is employed.

### Cation-exchange experiments

A dry NaCl–KCl salt melt was used for cation exchange. One feldspar plate together with a NaCl–KCl salt mixture was sealed into a quartz glass tube with an outer diameter of 9 mm and an inner diameter of 7 mm under vacuum. The salt mixture was applied in excess so that the molar amount of alkali cations in the salt melt was about 40 times the molar amount of alkali cations in the feldspar ensuring an essentially constant Na/K ratio in the salt melt during the cation-exchange experiments. Two different salt mixtures with $c^{\text {salt}}=0.25$ and $c^{\text {salt}}=0.30$, where $c ^{\text {salt}}= \mathrm{K}^{\text {salt}}/(\mathrm{K}^{\text {salt}} + \mathrm{Na}^{\text {salt}})$ (molar units), were used. Based on the experimentally determined partitioning of Na and K between NaCl–KCl salt melt and disordered alkali feldspar (Neusser et al. 2012), this corresponds to equilibrium compositions of the alkali feldspar of $c=0.50$ and $c=0.65$, respectively, at ambient pressure and $850\,^{\circ }\hbox {C}$. The maximum attainable compositional shift of the feldspar is thus $\Delta c = c^{\text {final}} - c^{\text {init}} = -\,0.35$, if the salt mixture with $c^{\text {salt}}= 0.25$ is applied, and $\Delta c= -\, 0.20$, if the salt mixture with $c^{\text {salt}}=0.30$ is applied. The feldspar-salt assemblies were annealed in a muffle furnace at $850\,^{\circ }\hbox {C}$ for 2, 4, 8, and 16 days. After annealing, the samples were quenched in cold water, to prevent diffusion-mediated modification of the Na–K distribution attained during the cation-exchange experiments. Finally, the tubes were opened, the salt was dissolved with deionized water, and the feldspar was retrieved. On the polished surfaces, fine parallel streaks marking the the cracks traces of the induced parallel cracks were visible. Mounts were prepared from the feldspar plates, which were cut perpendicular to the intersection lines between the cracks and the polished surfaces, so that the viewing direction in the microscope was parallel to both, the cracks and the polished surface the cracks emanated from. In this geometry, the angles between the cracks and the polished surface as well as the spacing between the cracks and the crack lengths are observed without distortion and in true length. The run conditions and the main characteristics of the run products are summarized in Table 1.

### Characterization of the run products

The polished sections of the run products were first documented at low magnification using an optical microscope. Higher magnification images were taken on a scanning electron microscope. Back scatter electron images were made on a Quanta 3D FEG instrument in the laboratory for electron-beam and ion-beam applications of the Faculty of Geosciences, Geography and Astronomy of the University of Vienna. Element distribution maps as well as quantitative point analyses were done on a CAMECA SXFive electron probe micro-analyzer in the same laboratory. An acceleration voltage of 15 kV and a beam current of 20 nA were applied for both element distribution maps and point analyses. Point analyses were made using a defocused beam to minimize loss of sodium by evaporation. Natural mineral standards were used as reference material for quantitative analyses.

## Results

**Fig. 2:**
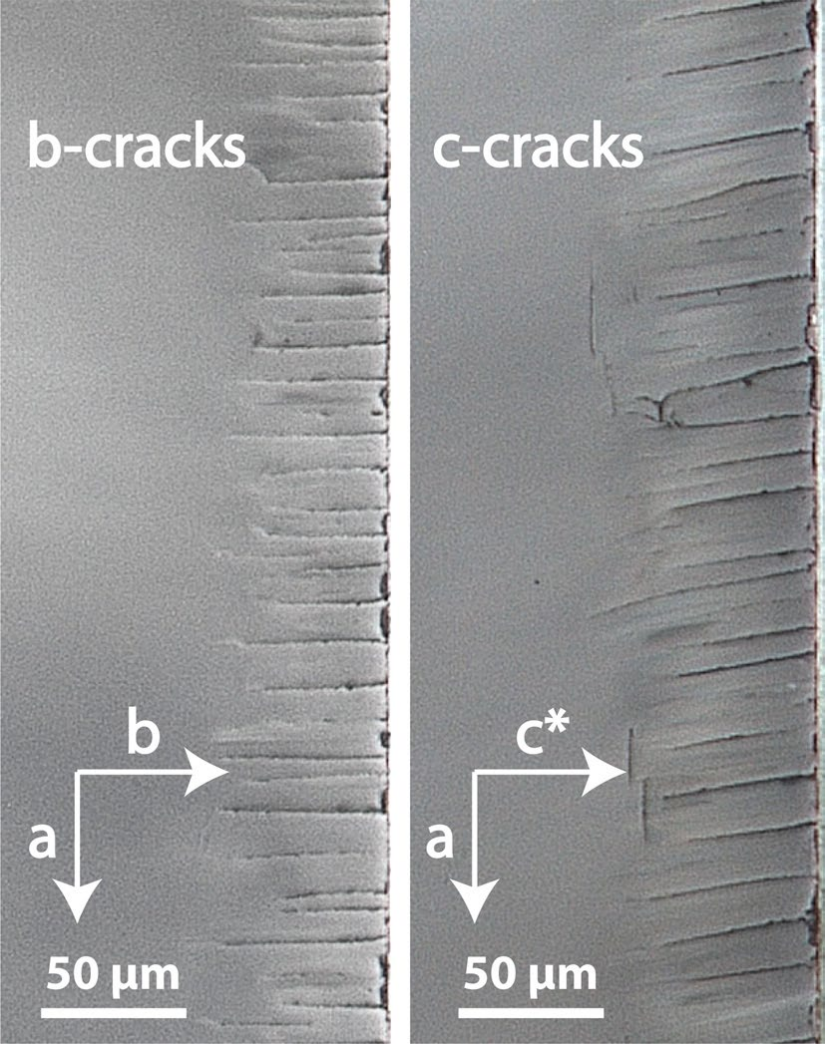
Reflected light photo-micrographs of b-cracks formed on the (010) (left) and of c-cracks formed on the (001) (right)-plates during cation exchange with an NaCl–KCl salt melt ($c^{\text {salt}}=0.25$) at $850\,^{\circ }\hbox {C}$ for 4 days

Parallel cracks were formed in all experiments. The cracks are observed in two different projections on the cross-sections of the (001)- and the (010)-crystal plates. On the cross-sections of the (001)-plates, cracks are observed emanating from the polished (001)-surfaces of the crystal plate and extending approximately parallel to the ${\mathbf {c}}$-direction; these cracks are referred to as “c-cracks”. On the cross-sections of the (010)-plates, cracks are observed emanating from the polished (010)-surfaces of the crystal plate and extending parallel to the ${\mathbf {b}}$-direction; these cracks are referred to as “b-cracks” (see Fig. 1). Actually, the b- and c-cracks are connected all around the crystal plate forming a single set of parallel cracks in 3D (Fig. 1). Nevertheless, for the following analysis we distinguish between two types of cracks, because always one type of cracks dominates on the different types of crystal plates. On the (001)-plates the c-cracks and on the (010)-plates the b-cracks dominate. They can be considered to develop to a large extent independent of one another. Reflected light images of sets of parallel b- and c-cracks are shown in Figure 2. As observed in an earlier study by Scheidl et al. (2013) the traces of the b-cracks enclose an angle of $90^\circ$ with the trace of the (010)-surface, and the traces of the c-cracks enclose an angle of $\approx 99^\circ$ with the trace of the (001)-surface, where the angle is measured from the trace of a crack extending approximately in [001]-direction towards the [100]-direction (Fig. 1). The cracks have a quite uniform spacing, which depends on the chemical shift applied to the feldspar. The crack spacing is about $15~ \upmu \hbox {m}$ for a chemical shift of $\Delta c = -\,0.35$, and it is about $40\,\upmu \hbox {m}$ for a chemical shift of $\Delta c = -\,0.20$. The cracks show quite uniform length for given run duration, salt composition and plate orientation (see Fig. 2). The characteristic crack lengths are given in Table 1.

**Table 1:** Crack spacing and length from 17 experimental runs; all experiments were done at $850\,^{\circ }\hbox {C}$ and close to ambient pressure; the values reported for crack length and spacings are mean values based on at least 30 individual measurements per sample

Sample	$c^{\text {salt}}$	Run duration (days)	Crack length ($\upmu \hbox {m}$)	Crack spacing ($\upmu \hbox {m}$)
(010)-25-6	0.25	6	71	19.25
(010)-25-8	0.25	8	95	21.52
(010)-25-16	0.25	16	170	22.64
(010)-30-2	0.30	2	31	
(010)-30-6	0.30	6	52	33.63
(010)-30-8	0.30	8	58	41.43
(010)-30-16	0.30	16	90	43.42
(001)-25-2	0.25	2	25	
(001)-25-4	0.25	4	49	
(001)-25-6	0.25	6	62	22.99
(001)-25-8	0.25	8	73	19.71
(001)-25-16	0.25	16	111	21.15
(001)-30-2	0.30	2	14	
(001)-30-4	0.30	4	29	
(001)-30-6	0.30	6	31	35.54
(001)-30-8	0.30	8	48	54.68
(001)-30-16	0.30	16	73	39.14

**Fig. 3:**
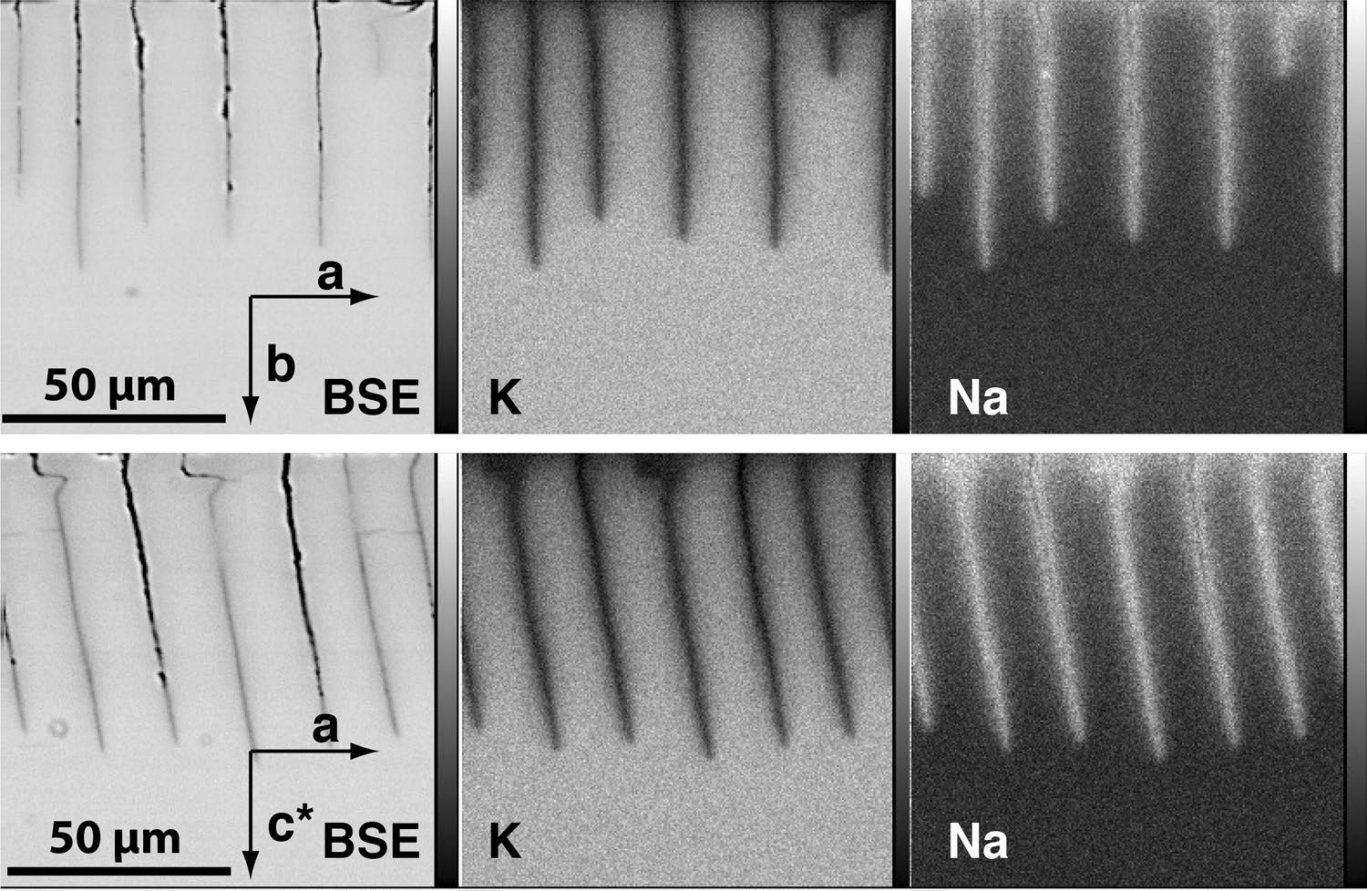
Top row: crack patterns formed on (010)-plates (b-cracks); bottom row: crack patterns formed on (001)-plates (c-cracks) during cation exchange with a NaCl–KCl salt melt ($c^{\text {salt}}=0.25$) at $850\,^{\circ }\hbox {C}$ for 4 days

BSE images and Na- as well as K-distribution maps of the exchanged feldspars are shown in Figure 3. All cracks are accompanied by a halo of slightly darker grey shade on the BSE images. Somewhat darker grey shades are also observed along the surfaces of the crystal plates. On the element distribution maps, these halos are identified as zones that are depleted in K and enriched in Na relative to the original alkali feldspar. The composition change from the internal, relatively K-rich to the external, more Na-rich zones is always gradual. There is no evidence of a discontinuity or a phase boundary that would indicate formation of a new phase due to reaction between feldspar and melt. The halos show a finger-like shape. They are symmetrical about the trace of the crack and point into the direction of crack propagation. The size and shape of the alteration halos around the crack tips are almost identical for different run durations indicating that the composition field was close to stationary, when referred to the position of the crack tip. The chemical alteration haloes along the crack flanks and around the crack tips indicate that the cracks formed during annealing at $850\,^{\circ }\hbox {C}$, concomitantly to Na–K cation exchange.

**Fig. 4:**
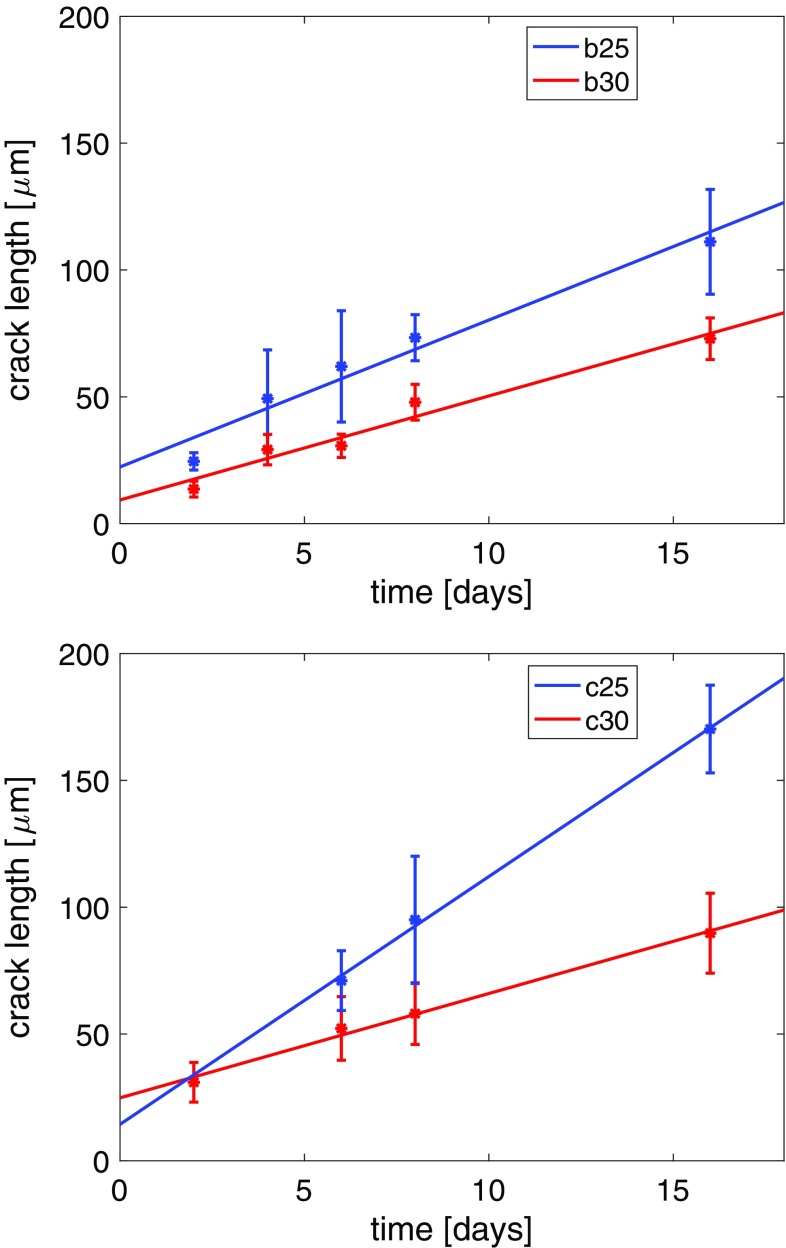
Crack length versus run duration; b25: b-cracks; the salt used for cation exchange had $c^{\text {salt}} = 0.25$; b30: same as b25 but produced by exchange with salt having $c^{\text {salt}} = 0.30$; c25 and c30: c-cracks from cation exchange with salts with $c^{\text {salt}} = 0.25$ and 0.30, respectively

Apart from the run duration, the crack length depends on the compositional shift applied to the feldspar and on the orientation of the crystal plate (see Fig. 4). For a given run duration and salt composition, the c-cracks are longer than the b-cracks. For given run duration and orientation of the crystal plate, the cracks are longer, if a compositional shift of $\Delta c = -\,0.35$ is applied compared to the cracks produced from a chemical shift of $\Delta c = -\,0.20$. For a given composition of the salt melt and orientation of the crystal plate, the crack length increases with increasing run duration (Fig. 4). Although there is considerable scatter in the measured crack length, the growth behaviour can be identified as approximately linear with respect to time. Linear extrapolations to $t = 0$ do not yield zero crack length, but they systematically produce a positive intercept on the ordinate axis.

## Discussion

### Crack formation

Parallel cracks are generated in the outermost portions of oriented plates of single-crystal, gem-quality alkali feldspars due to the shift of the feldspar from its original composition of $\mathrm{Na}_{0.15}\mathrm{K}_{0.85}\mathrm{AlSi}_3\mathrm{O}_8$ to a more sodium-rich composition during cation exchange with a NaCl–KCl salt melt of appropriate composition. The cation exchange occurs by the simultaneous in-diffusion of Na into and the out-diffusion of K from the outermost portions of the feldspar plate producing a Na-rich layer at the surface of the feldspar crystal. The *a*-, *b*-, and *c*-lattice parameters shrink with increasing Na-content, where the effect is largest for the *a*-parameter and substantially smaller for the *b*- and *c*-parameters (Kroll et al. 1986). Thus, during cation exchange the core region of the crystal plate still retains its original composition, while the surface layer has become more sodium-rich. Given that the core region and the surface layer interact as one solid, the associated eigenstrain in the surface layer (contraction primarily $\parallel {\mathbf {a}}$) with no eigenstrain in the volumetrically dominating, chemically unaltered core region of the crystal plate leads to a tensile stress component (approximately $\parallel {\mathbf {a}}$) in the “misfitting” surface layer. When the compositional shift in the chemically altered surface layer exceeds about 12 mole %, which corresponds to a tensile stress component of about 300 MPa in the surface layer, tensile (mode I) cracks form (Neusser et al. 2012; Scheidl et al. 2013). Two stages of fracturing may be discerned. The first stage cannot be observed directly with the ex-situ methods employed, but the following sequence of events is inferred: once a chemically altered surface layer has developed due to diffusion-mediated Na–K cation exchange between crystal and melt and the critical tensile stress of about 300 MPa has been reached, cracks nucleate. The cracks show nearly regular spacing, which is about $15\,\upmu \hbox {m}$ for a chemical shift of $\Delta c = -\,0.35$, and about $40\,\upmu \hbox {m}$ for a chemical shift of $\Delta c = -\,0.20$. The tensile stress component in the chemically altered surface layer is linearly related to the applied chemical shift and produces a crack spacing, which depends on the chemical shift. This indicates that crack formation is primarily controlled by the build-up of a tensile stress component in the chemically altered surface layer. Other effects such as surface roughness or other flaws in the crystal structure did not exert any measurable influence on the nucleation of cracks (Scheidl et al. 2013). It is hypothesized that, once nucleated, the cracks propagate into the sample nearly instantaneously. Thereby, they penetrate the chemically altered surface layer, but probably do not propagate far into the less altered or unaltered region beneath. The initial fracturing event reduces the tensile stress level in the chemically altered surface layer. Crack propagation comes to a halt as soon as the crack tip penetrates into the chemically less altered region, where the chemically induced eigenstrain and the associated tensile stress are too small to drive further crack propagation.

In the second stage, further crack propagation is driven by the chemical alteration that now also takes place along the cracks. Immediately after their formation, the cracks are infiltrated by the salt melt, and the crack flanks serve as new surfaces for cation exchange, and Na-rich/K-poor halos develop along the cracks. The associated eigenstrain causes a tensile stress state in the chemically altered halos driving further propagation of the cracks.

The alteration halos move into the interior of the crystal plate together with the propagating cracks. The formation of nearly stationary concentration fields around the crack tips allows assuming that the build-up of a tensile stress state due to successive diffusion-mediated cation exchange along the crack and at the crack tip and stress reduction by incremental propagation of the crack tip are intimately coupled. The rate of crack propagation is thus controlled by the efficiency of cation exchange along the crack and at the crack tip. This effect, in turn, is mediated by the interdiffusion of Na and K in the feldspar. The suggested interpretation of the crack development is corroborated by the fact that the c-cracks emanating from the (001)-surfaces and propagating into a [u0w]-direction at an angle of about $17^{\circ }$ from the [001]-direction measured towards the [100]-direction, grow faster than the b-cracks emanating from the (010)-surfaces and propagating into the [010]-direction. This crack-growth process is in line with the fact that Na/K interdiffusion is fast in the [u0w]-direction and slow in the [010]-direction (Petrishcheva et al. 2014; Schäffer et al. 2014). The intimate coupling between diffusion-mediated generation of an eigenstrain and associated stress field around the crack tip and crack propagation allows for estimating the critical energy release rate of crack propagation. In the next section, the eigenstrain- and stress fields at a propagating crack tip and the resulting energy release rate are calculated.

**Fig. 5:**
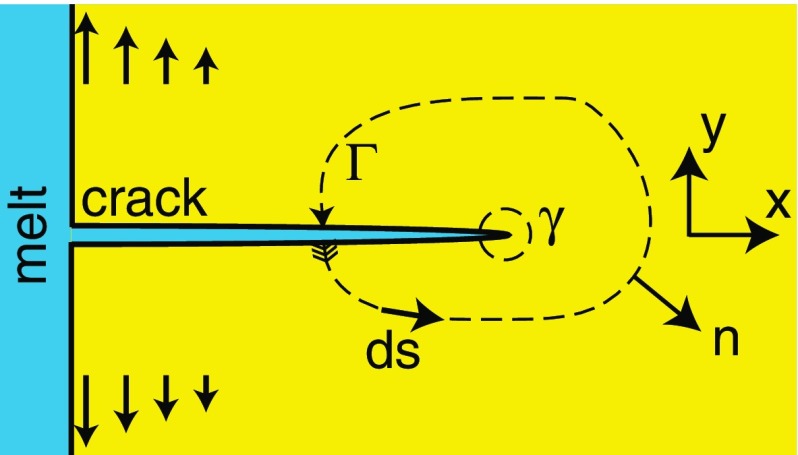
Schematic sketch of the J-integral

### Diffusion-induced crack propagation and energy release rate

#### The J-integral

The J-integral corresponds to the change in the total potential energy $\Pi$ supplied by the internal strain energy and by external forces of an elastic system with a change in the length $\ell$ of a crack.

$$\begin{aligned} J=-\frac{\mathrm{d} \Pi }{\mathrm{d}\ell }. \end{aligned}$$

If the planar geometry shown in Figure 5 is assumed, the J-integral can be quantified from the expression (Cherepanov 1967; Rice 1968)

1 $$\begin{aligned} J=\int _\Gamma \Bigl ( W\mathrm{d}y- \sum _{i,j=1}^2\sigma _{ij}n_i\partial _xu_j\mathrm{d}s \Bigr ), \end{aligned}$$

where *W*(*x*, *y*) is the strain energy density, $\pmb \sigma _{ij}(x,y)$ is the stress tensor, and ${\mathbf {u}}_j(x,y)$ is the displacement vector. The normal vector to the integration path $\Gamma$ is denoted by $\varvec{n}(x,y)$, and $\mathrm{d}s=|d\varvec{s}|$ is the length of an infinitesimal tangent vector along $\Gamma$. The J-integral is path independent for external loading of a homogeneous material, which is free of any eigenstrain (Cherepanov 1967; Rice 1968). In this case, an integration path around the crack tip can be replaced by the integration path $\Gamma$ as shown in Fig. 5. The local strain tensor is given as

$$\begin{aligned} \varepsilon _{ij} = \frac{1}{2}(\partial _iu_j+\partial _ju_i) \end{aligned}$$

and is composed of the eigenstrain tensor and the elastic strain tensor in a small displacement setting. If an inhomogeneous eigenstrain state prevails, the J-integral is path-dependent, for details see Simha et al. (2005). Nevertheless, the J-integral provides a reasonable estimate for the energy release rate of crack propagation, if the smallest integration path around the crack tip is chosen, which allows for numerically stable evaluation of the integral (Simha et al. 2003).

**Fig. 6:**
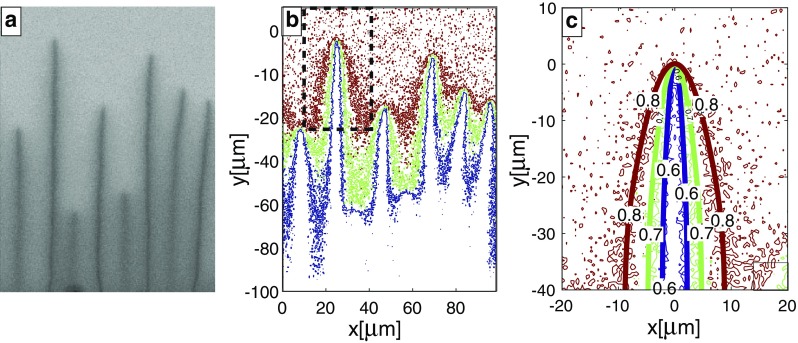
b-cracks after exchange with NaCl–KCl salt melt ($c^{\text {salt}}=0.25$) at $850\,^{\circ }\hbox {C}$ for 4 days; **a** K-distribution map; **b** Contours of $c = 0.6$, 0.7 and 0.8 obtained from the K-distribution map; **c** Blow up of the region indicated by the dashed rectangle in (**b**) showing contours obtained from the Na-distribution map (irregularly shaped curves) and from the 2D advection–diffusion model (smooth curves)

Practical application of Eq. (1) in case of an eigenstrain state requires that the position of the crack relative to the eigenstrain field is exactly known. Note that, in the case at hand, the eigenstrain field is related to the composition field via the composition dependence of the lattice parameters of the alkali feldspar (Kroll et al. 1986). The composition field around the crack is known from experimental data presented in the previous section. The available information is, however, too noisy (see Fig. 6) for a convincing calculation of the composition-induced eigenstrain, the resulting displacement field and its spatial derivative $\partial _x\varvec{u}(x,y)$. Furthermore, it must be noted that the crack lengths may have been altered during quenching and/or during the preparation of the polished cross-sections. The crack lengths observed on the polished cross-sections by means of optical- or scanning electron microscopy do not necessarily represent the crack length during the exchange experiment in every detail. In contrast to the crack length, the overall velocity of crack propagation is quite well constrained, because any random and systematic errors in the determination of the crack lengths, which could arise from modifications of the cracks occurring after the exchange experiment, cancel out. As a consequence, we follow an indirect procedure. We solve the diffusion problem numerically and fit the calculated concentration field to the experimental data. The J-integral is then evaluated using the numerical solution, which is smooth and where the position of the crack tip relative to the eigenstrain field is exactly known.

#### Composition field around a propagating crack

In a first step, the two-dimensional diffusion equation for the K-mole fraction $c=c(x,y,t)$

2 $$\begin{aligned} \partial _t c = D(\partial _x^2 c+\partial _y^2 c) \end{aligned}$$

is solved numerically in the domain shown in Fig. 5. Based on experimental evidence (Fig. 4), we postulate that the crack propagates at a constant velocity so that

$$\begin{aligned} \ell (t)=\ell _0 + vt, \end{aligned}$$

where $\ell _0$ is the initial crack length, and *v* is the velocity of crack propagation. The Na–K interdiffusion coefficient *D* is determined by fitting the experimental data shown in Fig. 6. It is useful to employ a moving frame (*X*, *Y*) by introducing the following coordinates:

$$\begin{aligned} X=x-\ell (t)=x-\ell _0-vt, \qquad Y=y. \end{aligned}$$

This transforms the diffusion Eq. (2) into the advection–diffusion equation

3 $$\begin{aligned} \partial _t C = v\partial _XC + D(\partial _X^2 C+\partial _Y^2 C) \end{aligned}$$

with $c(x,y,t)=C(X,Y,t)$. Equation (3) still applies to the domain shown in Fig. 5 but with a spatially fixed crack tip and a moving crystal. More specifically, we ignore the crack opening and associate the crack at time *t* with the interval

$$\begin{aligned} 0<x<\ell _0+vt \quad \text {and} \quad y=0 \quad \Leftrightarrow \quad -\ell _0-vt<X<0 \quad \text {and} \quad Y=0. \end{aligned}$$

Note that Eq. (3) refers to a moving frame and formally requires a moving boundary condition at the crystal–melt interface $X=-\ell _0-vt$. Numerics show that the diffusion process in the vicinity of the crack tip, which is the only process of interest, is practically independent of the crystal–melt boundary condition. The latter is, therefore, ignored by taking a characteristic crack length *L* and considering $X\in [-L,0]$ where

4 $$\begin{aligned} C(t,X,0)|_{-L<X<0} =C_0 \quad \text {and} \quad \partial _XC(t,X,Y)|_{X=-L} =0, \end{aligned}$$

and *L* is the final crack length. In summary, we do not consider the initial stage of crack formation. The cracks are already fully developed and filled with salt melt. Mineral-melt cation exchange occurs at the tip and at the flanks of the crack. It is known from earlier experiments (Neusser et al. 2012) that at $850\,^{\circ }\hbox {C}$ the surface of alkali feldspar attains the equilibrium composition with an NaCl–KCl salt melt within a few hours. It can thus be assumed that in our experiments the feldspar surfaces are in equilibrium with the salt melt, and the value of $C_0$ is given. The initial K-mole fraction in the interior of the crystal (the yellow domain in Fig. 5) is available. Consequently, Eq. (3) is provided with both, initial and boundary conditions. The above scheme can be generalized for a general $\mathbf {v}=(v_x,v_y)$.

**Fig. 7:**
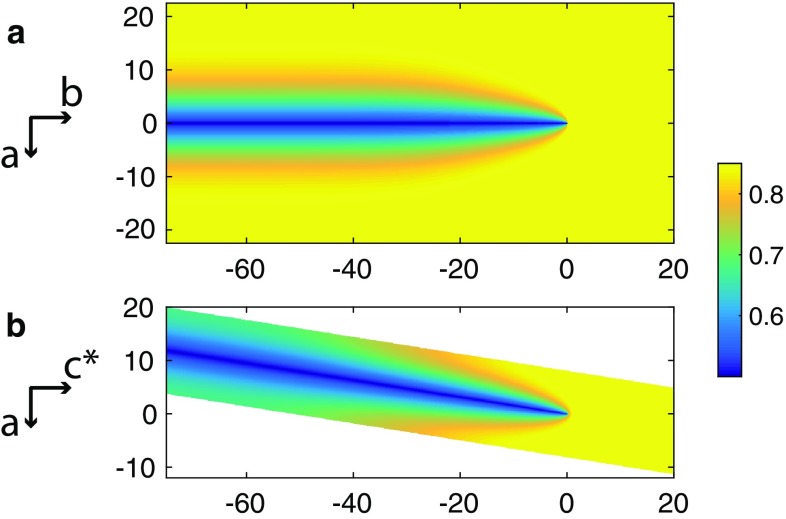
(**a**) Calculated concentration field in ${\mathbf {a}}$-${\mathbf {b}}$-section around the b-crack shown in Figure 6; the colour coding corresponds to the compositional range from $c = 0.5$ (blue) to 0.85 (yellow). The trace of the crack plane is parallel to the crystallographic ${\mathbf {b}}$-axis. (**b**) Calculated concentration field around a c-crack in ${\mathbf {a}}$-${\mathbf {c}}^*$-section. The trace of the crack plane encloses an angle of $9^\circ$ with the $c^*$-axis measured towards the [$\bar{1}00$]-direction

The velocity of crack propagation *v* was taken for the individual cracks that were used for fitting the concentration fields. For the b-crack $v= 8\,\upmu \hbox {m/day}$ and for the c-crack $v= 15\,\upmu \hbox {m/day}$. The only free parameter in Eq. (3) was the Na–K interdiffusion coefficient, *D*, which according to the diffusion anisotropy in alkali feldspar (Petrishcheva et al. 2014; Schäffer et al. 2014) takes the form

$$\begin{aligned} D_{ab} =\begin{pmatrix} D_{11} &{} 0 \\ 0 &{} D_{22} \\ \end{pmatrix} \quad \text {and} \quad D_{ac*} =\begin{pmatrix} D_{11} &{} D_{13} \\ D_{13} &{} D_{33} ,\\ \end{pmatrix} \end{aligned}$$

where $D_{ab}$ refers to the ${\mathbf {a}}$–${\mathbf {b}}$ plane, which is relevant for the b-cracks, and $D_{ac*}$ refers to the ${\mathbf {a}}$–${\mathbf {c}}$* plane, which is relevant for the c-cracks. The diffusivities were varied untill the numerical solutions fitted the experimentally observed composition fields around the cracks (see Fig. 6). For the b-cracks, the best agreement between calculated and observed concentration contours was obtained with $D_{11} = 4.8\cdot 10^{-5} \upmu \mathrm{m}^2 \mathrm{s}^{-1}$ and $D_{22} = 5.4\cdot 10^{-6} \upmu \mathrm{m}^2 \mathrm{s}^{-1}$ and for the *c*-cracks with $D_{11} = D_{13} = D_{33} = 4.8\cdot 10^{-5} \upmu \mathrm{m}^2 \mathrm{s}^{-1}$. For a b-crack, both experimental and numerical data are shown in Figure 6c. The Na–K interdiffusion coefficient in alkali feldspar at $850\,^{\circ }\hbox {C}$, including its composition and direction dependence, was determined experimentally by Petrishcheva et al. (2014) and by Schäffer et al. (2014) for $0.86 \le c \le 0.99$. In this compositional range *D* is a strictly monotonically increasing function of *c*. Whereas, the composition dependence is relatively modest at *c* = 0.86, it is comparatively strong at *c*= 0.99. The Na–K interdiffusion coefficient we obtained in our fitting procedure is by a factor of 0.3 lower than that given by Petrishcheva et al. (2014) and Schäffer et al. (2014) for $c = 0.86$ but with similar dependence on direction. In the light of the moderate composition dependence of *D*, this is reasonable in the compositional range of $0.50 \le c \le 0.85$ as in our experiments. The modelled composition fields around a b-crack and a c-crack for a compositional shift of $\Delta c = -\,0.35$, which was then used for calculating the eigenstrain field, are shown in Fig. 7. For a compositional shift of $\Delta c = -\,0.20$, the noise in the composition data is comparatively high, inducing an unacceptable uncertainty in numerical fitting. This is why the J-integral was only calculated for $\Delta c = -\,0.35$.

#### Eigenstrain field around a propagating crack

The eigenstrain field around a crack was calculated according to the concentration field as obtained from the above diffusion model using the composition dependence of the lattice parameters of alkali feldspar given by Kroll et al. (1986). A detailed description how a composition-dependent eigenstrain tensor of monoclinic alkali feldspar can be calculated from the crystallographic data of Kroll et al. (1986) can be found in Scheidl et al. (2013), and only a brief summary is given here. According to the monoclinic symmetry of the alkali feldspar the chemically induced eigenstrain tensor $\pmb \varepsilon ^{\text {chem}}_{ij}$ takes the form

$$\begin{aligned} \pmb \varepsilon ^{\text {chem}}_{ij}=\begin{pmatrix} \varepsilon ^{\text {chem}}_{11} &{} 0 &{} \varepsilon ^{\text {chem}}_{13} \\ 0 &{} \varepsilon ^{\text {chem}}_{22} &{} 0 \\ \varepsilon ^{\text {chem}}_{13} &{} 0 &{} \varepsilon ^{\text {chem}}_{33}\\ \end{pmatrix} . \end{aligned}$$

For calculating the compositionally induced eigenstrain the lattice parameters for the compositions $c = 0.84$ and $c = 0.69$ given by Kroll et al. (1986) were used, because this compositional range most closely reflects the compositional range of interest in our study. Based on these data, $\hat{\varepsilon }_{11}=-\,7.6\times 10^{-3}$, $\hat{\varepsilon }_{22}=-\,5.1\times 10^{-4}$, $\hat{\varepsilon }_{33}=-\,5.9\times 10^{-4}$, and $\hat{\varepsilon }_{13}=-\,1.9\times 10^{-3}$ are obtained for a compositional shift from $c = 0.84$ to $c = 0.69$, which corresponds to a compositional shift of $\Delta c = - \,0.15$ (Scheidl et al. 2013). The lattice parameters vary in a linear manner as a function of composition over the entire compositional range of interest (Kroll et al. 1986), so that the eigenstrain state for a composition *c* is obtained as

$$\begin{aligned} \varepsilon ^{\text {chem}}_{ij} = \frac{c-0.85}{-\,0.15} \cdot \hat{\varepsilon }_{ij}. \end{aligned}$$

#### Stress field around a propagating crack

For calculating the stress field, a local coordinate system was defined with the *X*-direction in the material *a*-direction and the *Y*-direction in the material *b*-direction for a b-crack, and with the *Y*-direction in the material $c^*$-direction for a c-crack. The *Z*-direction is normal to the *X*–*Y* plane. A strip with the width of one crack distance in the *X*-direction and $500~ \upmu \hbox {m}$ in the *Y*-direction (that is half the thickness of the test specimen) was defined as a planar simulation cell. The parallel side flanks, together with the top and bottom, form the simulation cell as a rectangle for a b-crack and a parallelogram (inclination angle $99^{\circ }$) for a c-crack. The crack starts in the middle of the top and extends parallel to the side flanks towards the bottom of the simulation cell. Periodic boundary conditions were assumed at both sides. The simulation cell was fixed at the left bottom corner and allowed to move only in the *X*-direction at the right bottom corner. Generalized plane strain for the 2D-configuration was applied, implying that the displacement of the whole simulation cell in the *Z*-direction assumes the same value in all points, with the side condition that no resultant force in the *Z*-direction acts on the unit cell.

**Fig. 8:**
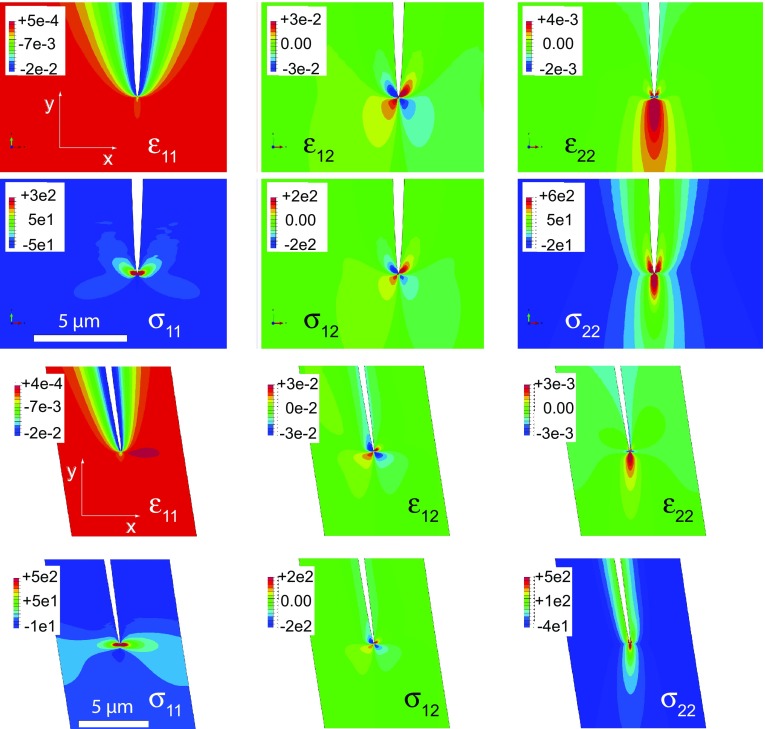
Calculated stress (MPa) and strain fields in the vicinity of the tips of a b-crack (two top rows) and a c-crack (two bottom rows) after exchange with NaCl–KCl salt melt ($c^{\text {salt}}=0.25$) at $850\,^{\circ }\hbox {C}$ that have been propagating with $v= 8\,\upmu \hbox {m/day}$ and $v= 15\,\upmu \hbox {m/day}$, respectively for 4 days

The elasticity tensor $\mathbf {L}$ is formulated with respect to the global *X*, *Y*, and *Z* system. In the according tensor/vector notation *X*, *Y*, and *Z* are assigned to 1, 2, and 3, respectively. The elasticity tensor $\mathbf {L}$ is arranged with the data from Haussuehl (1993) as a $6\times 6$ tensor relating the stress vector $\pmb \sigma ^T = \left( \sigma _{11}, \sigma _{22}, \sigma _{33}, \sigma _{12}, \sigma _{13}, \sigma _{23} \right)$ to the strain vector $\pmb \epsilon ^T = \left( \epsilon _{11}, \epsilon _{22}, \epsilon _{33}, \epsilon _{12}, \epsilon _{13}, \epsilon _{23} \right)$. About $10^5$ linear four-node elements with a dense mesh near the crack tip and along the crack and a less dense mesh along the side flanks of the simulation cell were applied. The calculated strain and stress fields around the tips of a–b and a–c crack are shown in Fig. 8.

#### Calculation of the near-tip J-integral

The J-integral was evaluated for both, the b-crack and the c-crack, and for the compositional shift $\Delta c = -\,0.35$. For simplicity, all cracks of the same type were assumed to have the same length and spacing. This allows to solve Eq. (3) just for one crack. The integration domain along *OY* corresponds to the distance between uniformly spaced cracks. The resulting values of the near-tip J-integral are $-\,1.8$ and $-\,2.2\,\hbox {J}\,\hbox {m}^{-2}$ for the b-crack and the c-crack, respectively. Based on the notion of the intimate coupling among chemical alteration and associated eigenstrain, stress build-up and stress reduction by incremental fracturing, the value of the J-integral can be interpreted as the critical energy release rate that is required for propagation of the crack. Although b-crack and c-crack experiments are completely independent of one another, both the b- and c-cracks have the same orientation with respect to the crystal lattice. As a consequence, the energy release rates obtained from the analysis of the b-cracks and the c-cracks are expected to be similar. The observed difference of about 20% between the two values reflects the internal reproducibility of the determination.

For ideal brittle fracturing the plastic deformation at the crack tip is negligible, and the energy release rate of fracturing corresponds to twice the surface energy $\gamma$. Although plastic deformation in feldspar is well known from deformation at high confining pressure in natural and synthetic systems, it is unlikely to play a significant role at the conditions and time scales of our experiments. As a consequence, the J-integral provides a good estimate for the surface energy, which is inferred to be $\gamma = 0.9$–$1.1\,\hbox {J}\,\hbox {m}^{-2}$. It is important to note, that the b- and c-cracks have the same orientation with respect to the lattice of the feldspar crystal. The crack orientation does not correspond to any low-index lattice plane. It rather represents a general feldspar surface. Our determination of the energy release rate and of the corresponding surface energy of sanidine falls well into the range of the values given by Tromans and Meech (2002). Using a microscopic approach based on the Born model for bonding in ionic crystals, the latter authors obtained critical energy release rates ranging from 0.676 to $20.75\,\hbox {J}\,\hbox {m}^{-2}$ and according surface energies ranging from 0.338 to $10.33\,\hbox {J}\,\hbox {m}^{-2}$ for a range of rock-forming minerals and minerals of economic interest. They report a critical energy release rate of $5.48\,\hbox {J}\,\hbox {m}^{-2}$ for anorthite feldspar and a corresponding surface energy of $2.74\,\hbox {J}\,\hbox {m}^{-2}$, which is by a factor of 2.5–3 higher than values obtained in the present study.

Failure of minerals under load was investigated using indentation experiments (Broz et al. 2006; Whitney et al. 2007), where the critical stress intensity factor $K_\mathrm{Ic}$, which is also referred to as the fracture toughness, is determined from the length of radial cracks emanating from the corners of an indentation mark produced with a Vickers indenter. The fracture toughness of orthoclase was determined as $0.88\,\hbox {MPa}\,\hbox {m}^{1/2}$ (Broz et al. 2006) and $1.1\,\hbox {MPa}\,\hbox {m}^{1/2}$ (Whitney et al. 2007). Bernardo et al. (2008) reported an indentation fracture toughness of $1.5\,\hbox {MPa}\,\hbox {m}^{1/2}$ for sintered orthoclase–microcline ceramics. The critical energy release rate $G_\mathrm{Ic}$ can be calculated from the critical stress intensity factor $K_\mathrm{Ic}$ accordingly as

5 $$\begin{aligned} J = G_\mathrm{Ic } = \frac{K_\mathrm{Ic}^2}{\bar{E}}. \end{aligned}$$

Note that $\bar{E}$ is the plane strain bulk modulus, $\bar{E} = E/(1-\nu ^2)$, with *E* being Young’s modulus and $\nu$ the Poisson’s ratio, see Gross and Seelig (2011) for details. The critical energy release rates obtained from the critical stress intensity factors of Broz et al. (2006) and Whitney et al. (2007) using Eq. (5) are 7.2 and $11.2\,\hbox {J}\,\hbox {m}^{-2}$, respectively. These values are somewhat higher than the estimation obtained from our study. There are several potential reasons for this discrepancy. The cracks produced during indentation experiments have a complex 3D geometry, and the determination of $K_\mathrm{Ic}$ is based on an empirical relation between the length of radial cracks from indentation, the applied load, the indentation hardness, and the elastic modulus (Lawn et al. 1980). This relation does not correspond to the continuum mechanical definition of energy release rate. Comparison of the numerical values must thus be done with caution. Moreover, the orthoclase used by Broz et al. (2006) and by Whitney et al. (2007) was perthitic and not of gem-quality like the material used in our study. The critical stress intensity factor of $K_\mathrm{Ic}= 2.3$–$2.7\,\hbox {MPa}\,\hbox {m}^{1/2}$ given by Scheidl et al. (2013) is probably overestimated, because the latter authors did not consider the chemical alteration and associated stress state along the cracks. However, their estimation of the critical stress needed for fracturing and the relation between the extent of chemical shift and the characteristic crack spacing are valid.

#### Feldspar replacement mediated by chemically induced fracturing

Orthoclase (or sanidine), mantled by sodium-rich plagioclase, is a typical example of high-temperature feldspar alteration, which often accompanies felsic–mafic melt interaction in plutonic and volcanic environments (Stimac and Wark 1992; Ginibre et al. 2007). Naturally occurring mantled feldspars (Stimac and Wark 1992; Ginibre et al. 2007) as well as mantled feldspar produced in piston cylinder experiments (Wark and Stimac 1992) show a system of parallel cracks, referred to as “dissolution channels”. These cracks penetrate into the alkali feldspar ahead of the actual replacement front and act as nucleation sites for blades of newly forming sodium-rich plagioclase. In natural feldspar replacement, a diffusion front develops ahead of the actual felspar replacement front. There the in-diffusion of Na into the K-rich feldspar produces a tensile stress state, which eventually induces fracturing. In contrast to our cation-exchange experiments, where the only effect of chemical alteration of the reactant feldspar is the formation of cracks, in natural feldspar replacement the metasomatizing agent, be it a fluid or a melt, induces precipitation of new sodium-rich feldspar in addition. However, like in our cation-exchange experiments, the overall conversion of orthoclase or sanidine phenocrystals to sodium-rich plagioclase appears to be controlled by the successive propagation of cracks into the interior of the precursor grain. Our cation-exchange experiments show how diffusion-controlled chemical alteration, eigenstrain state, stress state and crack formation are coupled.

## Conclusions

A system of parallel cracks was produced in monoclinic potassium-rich gem-quality alkali felspar by a shift towards more sodium-rich composition generated by means of cation exchange with a NaCl–KCl salt melt at a temperature of $850\,^{\circ }\hbox {C}$ and close to ambient pressure. Cation exchange occurred due to the simultaneous in-diffusion of sodium and the out-diffusion of potassium, which yielded a layer of chemically altered feldspar at the surface of the specimen. Two stages of fracturing may be discerned. The first stage is characterized by the successive build-up of a tensile stress state in the chemically altered surface layer, which results from the shrinkage of the lattice parameters with increasing sodium content. Once the induced stress level exceeds the tensile strength of the felspar, fracturing occurs. The chemically induced eigenstrain is highly anisotropic. It is largest for the *a*-parameter and by a factor of about 5 smaller for the *b*- and *c*-parameters. Accordingly, the cracks are oriented approximately perpendicular to the *a*-direction. The cracks show a characteristic spacing, which decreases with an increase of the applied compositional shift. Once nucleated, the cracks propagate nearly instantaneously through the chemically altered surface layer, but come to a halt in the unaltered feldspar beneath, where no driving force for further fracturing is available.

The second stage of fracturing is characterized by continuous crack propagation. Once formed, the cracks are infiltrated by the salt melt, and the crack flanks provide new surfaces for cation exchange. Finger-shaped halos of chemical alteration form along the cracks and around the crack tips. The resulting build-up of a tensile stress state around the crack tips is balanced by the successive propagation of the cracks. Then nearly stationary composition and eigenstrain fields as well as stress states develop around the crack tips and travel into the interior of the crystal together with the cracks. As a consequence, the cracks extend with nearly constant velocity. The critical energy release rate of fracturing obtained via evaluating the near-tip J-integral has a value of 1.8–$2.2\,\hbox {J}\,\hbox {m}^{-2}$. The corresponding surface energy of the crack flanks, which correspond to a general plane in the sanidine, is $\le 0.9$–$1.1\,\hbox {J}\,\hbox {m}^{-2}$.

The mechanism of diffusion-controlled crack propagation leads to a positive feedback between chemical alteration and fracturing. It provides an efficient pathway for the alteration of feldspar at high temperatures such as the replacement of sanidine and orthoclase by sodium-rich plagioclase in magmatic environments.

## References

[CR1] Abart R, Petrishcheva E, Wirth R, Rhede D (2009). Exsolution by spinodal decomposition II: perthite formation during slow cooling of anatexites from Ngoronghoro, Tanzania. Am J Sci.

[CR2] Angel R, Sochalski-Kolbus LM, Tribaudino M (2012). Tilts and tetrahedra: the origin of the anisotropy of feldspars. Am Mineral.

[CR3] Bazant ZP, Ohtsubo H (1977). Stability conditions for propagation of a system of cracks in a brittle solid. Mech Res Commun.

[CR4] Bazant ZP, Ohtsubo H, Aoh K (1979). Stability and post-critical growth of a system of cooling or shrinkage cracks. Int J Fract.

[CR5] Bernardo E, Doyle J, Hampshire S (2008). Sintered feldspar glass-ceramics and glass-ceramic matrix composites. Ceram Int.

[CR6] Broz ME, Cook RF, Whitney DL (2006). Microhardness, toughness, and modulus of mohs scale minerals. Am Mineral.

[CR7] Cherepanov GP (1967). The propagation of cracks in a continuous medium. J Appl Math Mech.

[CR8] Cherniak DJ (2010). Cation diffusion in feldspars. Rev Mineral Geochem.

[CR9] Demtröder K (2011) Untersuchung zur Al/Si-Ordnung an Sanidin Megakristallen aus der Eifel. Master thesis, Ruhr-Universität Bochum

[CR10] Ginibre C, Woerner G, Kronz A (2007). Crystal zoning as an archive for magma evolution. Elements.

[CR11] Gross D, Seelig T (2011). Fracture mechanics with an introduction to micromechanics.

[CR12] Haussuehl S (1993). Thermoelastic properties of beryl, topaz, diaspore, sanidine and periclase. Zeitschrift für Kristallographie.

[CR13] Keer LM, Nemat-Nasser S, Oranratnachai A (1979). Unstable growth of thermally induced interacting cracks in brittle solids—further results. Int J Solids Struct.

[CR14] Kroll H, Ribbe PH (1983) Feldspar mineralogy, volume 2 of reviews in mineralog, chapter lattice parameters, composition and Al,Si order in alkali feldspars. Mineralogical Society of America, pp 57–100

[CR15] Kroll H, Schmiemann I, von Coelln G (1986). Alkali feldspar solid-solutions. Am Mineral.

[CR16] Lawn BR, Evans AG, Marshall DB (1980). Elastic/plastic indentation damage in ceramics: the median/radial crack system. J Am Ceram Soc.

[CR17] Neusser G, Abart R, Fischer F-D, Harlov D, Norberg N (2012). Experimental Na/K exchange between alkali feldspar and an NaCl-KCl salt melt: chemically induced fracturing and element partitioning. Contrib Mineral Petrol.

[CR18] Parker AP (1999). Stability of arrays of multiple edge cracks. Eng Fract Mech.

[CR19] Petrishcheva E, Abart R (2009). Exsolution by spinodal decomposition I: evolution equation for binary mineral solutions with anisotropic interfacial energy. Am J Sci.

[CR20] Petrishcheva E, Abart R (2012). Exsolution by spinodal decomposition in multicomponent mineral solutions. Acta Mater.

[CR21] Petrishcheva E, Abart R, Schäffer A-K, Habler G, Rhede D (2014). Sodium-potassium interdiffusion in potassium-rich alkali feldspar I: full diffusivity tensor at $$850^\circ \text{C}$$. Am J Sci.

[CR22] Ribbe PH (1983). Chemistry, structure and nomenclature of feldspars.

[CR23] Rice JR (1968). A path independent integral and the approximate analysis of strain concentration by notches and cracks. J Appl Mech.

[CR24] Schäffer A-K, Petrishcheva E, Habler G, Abart R, Rhede D, Giester G (2014). Sodium-potassium interdiffusion in potassium-rich alkali feldspar II: composition- and temperature-dependence obtained from cation exchange experiments. Am J Sci.

[CR25] Scheidl K, Schaeffer AK, Petrishcheva E, Habler G, Fischer F-D, Schreuer J, Abart R (2013). Chemically induced fracturing in alkali feldspar. Phys Chem Miner.

[CR26] Simha NK, Fischer F-D, Kolednik O, Chen CR (2003). Inhomogeneity effects on the crack driving force in elastic and elastic-plastic materials. J Mech Phys Solids.

[CR27] Simha NK, Kolednik O, Fischer F-D (2005). Material force models for crack—influences of eigenstrains, thermal strains & residual stresses. Proc 11th Int Conf Fract Topic.

[CR28] Stimac JA, Wark DA (1992). Plagioclase mantles on sanidine in silicic lavas, Clear-Lake, California—implications for the origin of Rapakivi texture. Geol Soc Am Bull.

[CR29] Sumi Y, Nemat-Nasser S, Keer LM (1980). A new combined analytical and finite-element solution method for stability analysis of the growth of interacting tension cracks in brittle solids. Int J Eng Sci.

[CR30] Tromans D, Meech JA (2002). Fracture toughness and surface energies of minerals: theoretical estimates for oxides, sulphides, silicates and halides. Miner Eng.

[CR31] Wark DA, Stimac JA (1992). Origin of mantled (Rapakivi) feldspars—experimental-evidence of a dissolution-controlled and diffusion-controlled mechanism. Contrib Mineral Petrol.

[CR32] Whitney DL, Broz M, Cook RF (2007). Physical properties of metamorphic minerals. Am Mineral.

[CR33] Yund R (1984). Feldspars and feldspathoids. Alkali feldspar exsolution: kinetics and dependence on alkali interdiffusion.

